# Neutrophilic Dermatosis of the Dorsal Hands: Report of a Case and Review of the Literature

**DOI:** 10.1155/2019/8301585

**Published:** 2019-01-22

**Authors:** Narciss Mobini, Kaviyon Sadrolashrafi, Susun Michaels

**Affiliations:** ^1^University of Nevada, School of Medicine, Reno and Las Vegas, NV, USA; ^2^Director of Dermatopathology, Associated Pathologists, Chartered, Las Vegas, NV, USA; ^3^4230 Burnham Ave., Las Vegas, NV, 89119, USA; ^4^University of California, Los Angeles (UCLA), Department of Life Sciences, Los Angeles, CA, USA; ^5^Touro College of Osteopathic Medicine, Las Vegas, NV, USA; ^6^Las Vegas Skin and Cancer Clinics, Las Vegas, NV, USA

## Abstract

Neutrophilic dermatosis of the dorsal hands is an underrecognized entity, which is a distributional variant of Sweet's syndrome. It is often clinically misdiagnosed as an infectious process in overwhelming majority of the cases and the treatment is therefore delayed. Also, its association with underlying systemic and neoplastic disorders makes the need for an accurate diagnosis more crucial. We present a 45-year-old Caucasian woman who was initially diagnosed as having a hand infection with unsuccessful courses of antibiotic therapy. A later biopsy revealed a diffuse dermal infiltrate of neutrophils with leukocytoclasis, vasculopathic changes, and marked papillary dermal edema. Patient responded rapidly to oral prednisone treatment. By sharing a new case and comprehensive review of available published literature, we intend to raise awareness of this underreported entity and emphasize the role of timely biopsy of the lesions that will not only lead to an accurate diagnosis, but also avoid unnecessary antibiotic treatments, potentially aggressive management strategies such as surgical debridement or amputation, and referrals to wound care centers. More importantly, it will prompt a search to exclude any possible association, particularly hematopoietic malignancies.

## 1. Introduction

Pustular vasculitis of the hands was first introduced by Strutton et al. in 1995, to describe an eruption on the dorsal hands resembling Sweet's syndrome, but showing leukocytoclastic vasculitis histologically [1]. Later, clinically similar cases were reported with no vasculitis [2–4]. Neutrophilic dermatosis of the dorsal hands (NDDH), proposed by Galaria et al. in 2000 is currently the widely accepted term for this clinicopathologic entity [4]. We present a new case of NDDH in a woman who was originally misdiagnosed as having an infection.

## 2. Case Report

**Figure 1:**
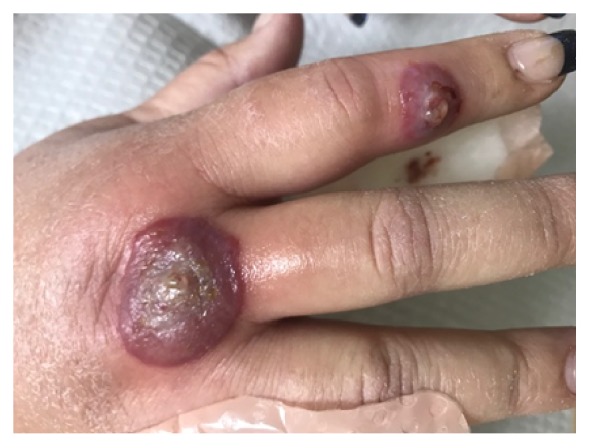
Centrally ulcerated edematous nodules with violaceous borders on the dorsal aspects of right index finger and proximal metacarpophalangeal joint of mainly third finger. Note the bulbous edema of the affected digits.

**Figure 2:**
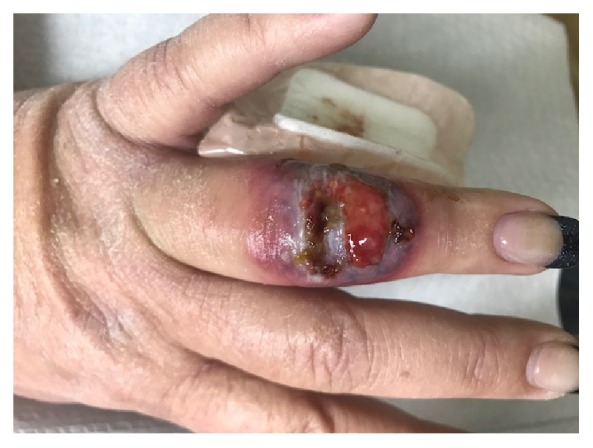
Large ulcerated oozing plaque on the dorsum of left ring finger with purulent-appearing exudate.

**Figure 3:**
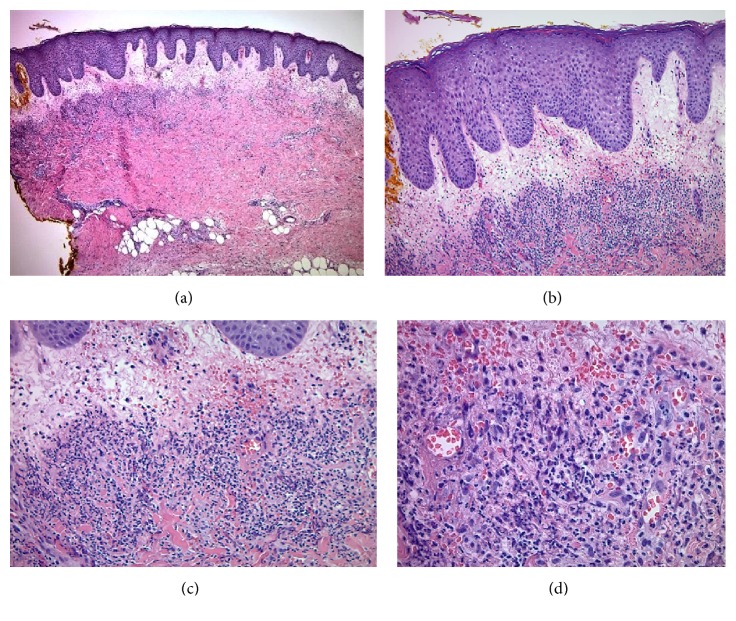
(a,b,c,d) Marked papillary dermal edema, superficial and deep perivascular, interstitial, and diffuse infiltrate of neutrophils with leukocytoclasia, extravasation of erythrocytes, vasculopathic changes, and absence of true vasculitis (hematoxylin-eosin 4X, 10X, 20X, and 40X, respectively).

**Figure 4:**
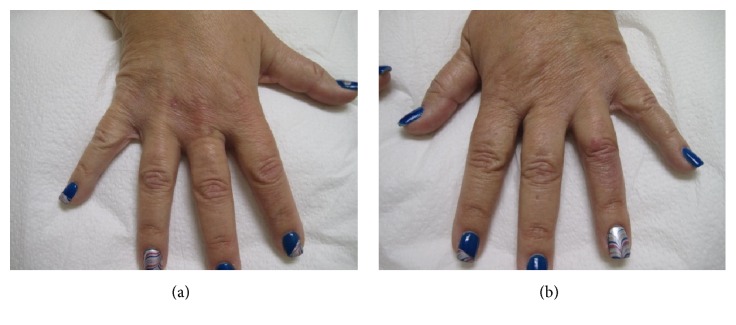
(a,b) Healed lesions with subtle residual erythematous patches, four weeks after treatment with oral prednisone.

A 45-year-old Caucasian obese woman presented with small painful ulcers on the back of her hands and fingers that had started three weeks prior to her visit. She first noticed small red flat discolored areas which gradually worsened by developing pain, swelling, and ulcers within two weeks. She did not recall a prior trauma. She had a history of previous laparoscopic gastric sleeve surgery for morbid obesity and a vague diagnosis of mild diabetes for which she was not on any medication. She denied taking a new drug. With the clinical diagnosis of an infectious process, bacterial culture and sensitivity were performed and she was given oral and topical antibiotics (Bactrim and mupirocin, respectively) along with wound care instructions. The patient started developing fever with malaise and was admitted to the emergency room, where she was placed on intravenous antibiotic (vancomycin) due to suspicion of sepsis, originating from her “hand infection”. After a few days, she returned to our clinic. Compared to the original visit, the condition appeared worse with development of erythematous ulcerated nodules and plaques, violaceous borders, and marked surrounding edema, present on the dorsal aspects of right index and left ring fingers along with proximal metacarpophalangeal joint of third digit. The fingers in the nonulcerated areas displayed a fusiform swelling (Figures 1 and 2). She also developed tender indurated erythematous plaques on the dorsum of the right wrist. Examination of the rest of the body, including the mucosal surfaces, failed to show any involvement. Based on the clinical progression and lack of response to antibiotics, biopsy was obtained to rule out atypical pyoderma gangrenosum (PG), deep fungal or mycobacterial infection, or other possibilities. Histopathologic examination revealed marked subepidermal edema associated with a superficial and deep perivascular, interstitial, and diffuse infiltrate of neutrophils, many of which were present within the vessel walls, associated with leukocytoclasia and extravasation of erythrocytes. Despite vasculopathic changes, there was no evidence of true vasculitis (Figures 3(a), 3(b), 3(c) and 3(d)). Although the histopathologic differential diagnosis was most consistent with Sweet's syndrome, based on the clinical presentation, NDDH was the rendered diagnosis. She was immediately started on oral prednisone 80mg per day. In the meantime, a systemic workup was carried out. After a week, the ulcers were already healing and the swelling was subsiding. Tissue cultures yielded negative results and systemic workup was normal. We started to taper down the prednisone at this point. One week later, there was continued flattening of the lesions. After one month, there was mild residual erythema at the previous sites (Figures 4(a) and 4(b)). We continued to taper down her prednisone and prescribed a potent topical corticosteroid in case of local recurrence. She reported complete clearing of the lesions. Seven months later, she presented with a mildly erythematous patch on the dorsum of right third digit. An intralesional steroid injection was performed followed by topical steroids for two weeks. She has not had any recurrence, after one year from the original incidence.

## 3. Discussion

The term neutrophilic dermatosis of the dorsal hands (NDDH) was first proposed by Galaria et al. in 2000, who considered it to be a subset of Sweet's syndrome [4]. A thorough review of the literature reveals that only close to 100 cases have so far been reported [5–10]. Today, NDDH is most widely accepted to represent a ‘distributional or localized variant' of Sweet's syndrome which belongs to the spectrum of neutrophilic dermatoses, rather than a primary vasculitis and that any vasculitis seen histologically is a secondary phenomenon [11, 12]. It is likely that the timing of the biopsy during the evolutionary phases of the lesions may result in different findings with regard to presence or absence of vasculitis. Clinically, the lesions are characterized by painful erythematous and violaceous papules, plaques, nodules, pustules, and hemorrhagic bullae. These may eventually ulcerate in 50% of the cases. The most common sites of eruption are the dorsal aspect of both hands. Also, back of fingers or wrists can be affected with rare involvement of the palms. There may be concurrent or subsequent lesions elsewhere, such as back, lips, legs, and forehead [5, 8, 13–16]. Women are affected more than men, comprising 70% of the cases [7]. In one series, up to 65% of cases reported a proceeding trauma, which may be misleading [10]. Fever is mentioned in 33% of the reports [7]. The most common clinical differential diagnosis is an infection, for which the patients receive antibiotics, with no success. Deep fungal, atypical mycobacterial, parasitic, and viral infections may also be considered clinically. In the noninfectious category, atypical PG, bullous Sweet's or Sweet's-like syndrome, bullous erythema multiforme, or a pustular drug reaction are in the differential diagnosis [6]. Although rather deep ulceration with undermining edges is common in classic PG, a superficial ulcer with hemorrhagic bullae is more commonly seen in atypical PG or bullous Sweet's syndrome. On the other hand, ulceration is uncommon in typical Sweet's syndrome and, if present, is suggestive of an underlying hematologic malignancy. It is suggested that many cases diagnosed as atypical Sweet syndrome, atypical PG, or PG-Sweet overlap, in fact represent NDDH, when manifesting in this distinctive anatomic distribution [7, 17]. Histologically, there is prominent papillary dermal edema, superficial and deep perivascular, and diffuse infiltrate of neutrophils with leukocytoclasia, extravasated erythrocytes, and no vasculitis. Admixed lymphocytes and occasional eosinophils can also be seen. It appears that any vasculitis observed, is of secondary type rather than a primary phenomenon, similar to PG or Sweet's. Occasionally one may see epidermal changes such as spongiosis, neutrophilic microabscesses, or pseudoepitheliomatous hyperplasia [5, 9, 10]. The most important associations with NDDH are neoplastic disorders in 27% of cases, where it may represent a paraneoplastic phenomenon. The most common are hematologic disorders, such as myelodysplastic syndrome, acute leukemia, lymphoma, or other diseases in 21% of cases [7, 17–19]. In addition, solid neoplasms can be seen such as cancers of breast, kidney, colon, stomach, lung, and hypopharynx [1, 2, 7, 19–22]. Associated nonneoplastic disorders include inflammatory bowel disease, in approximately 15% of cases [7, 17, 22]. Diverticulosis, diverticulitis, acute proctitis, and history of small bowel obstruction or bypass have also been reported [5]. In bowel-associated dermatosis-arthritis syndrome, the skin lesions develop in patients with prior bowel bypass surgeries and other bowel disorders; however, the cutaneous eruption is more widespread, involving the upper extremities and trunk. Classic PG is seen with increased incidence of inflammatory bowel disorders as well; however, the distribution of lesions is different. Our patient had a history of laparoscopic sleeve gastrectomy for morbid obesity in the past. Other conditions reported in NDDH include diabetes mellitus, peripheral ulcerative keratitis, erythema nodosum, sarcoidosis, chronic hepatitis C, urinary tract infection, streptococcal tonsillitis, and chronic glomerulonephritis [5, 6, 23–26]. There is a report of NDDH after exposure to a chemical fertilizer containing ammonium nitrate and calcium salts [27]. An insect bite has been proposed as possible culprit of NDDH through a pathergic reaction, which occurred in a unilateral distribution [28]. Among drug-induced cases, thalidomide and its analogue lenalidomide are the main reported agents [29, 30]. NDDH occurring after chemotherapy for AML has also been reported [31]. There are many occasions that no underlying cause can be found [12, 32]. The mainstay of treatment is oral corticosteroids, with strikingly rapid response. Some have included dapsone, colchicine, minocycline, or pentoxifylline [4, 5, 7, 17, 25, 32]. There may be recurrences as high as 10% [20]. In our patient, after complete healing of the lesions, there was a mild recurrence seven months later, treated with an intralesional injection of Kenalog, followed by topical steroids for two weeks. No further recurrence has been noted in about a year from her original visit.

## 4. Conclusion

NDDH is best regarded as a distributional variant of Sweet's syndrome, where the lesions occur on the dorsal aspects of both hands in its typical presentation. In the majority of cases, the initial clinical diagnosis is an infectious process. In addition, patients are referred to wound care centers due to nonhealing wounds. Therefore, unsuccessful antibiotic treatments, failed surgical debridements, and even amputations could follow. By presenting a new case of NDDH and review of the existing published data, we intend to raise awareness of this clinicopathologic entity for dermatology practitioners and dermatopathologists. NDDH should be strongly considered in lesions occurring on the dorsal hands, particularly if suspicious of infection, and timely biopsy be performed. In addition, recognizing this disease should prompt the clinician for a thorough investigation to rule out any associated malignancy or systemic disorder.

## References

[1] Strutton G., Weedon D., Robertson I. (1995). Pustular vasculitis of the hand. *Journal of the American Academy of Dermatology*.

[2] Curcó N., Pagerols X., Tarroch X., Vives P. (1998). Pustular vasculitis of the hands. Report of two men. *Dermatology*.

[3] Hall A. P., Goudge R. J., Ireton H. J., Burrell L. M. (1999). Pustular vasculitis of the hands. *Australasian Journal of Dermatology*.

[4] Galaria N. A., Junkins-Hopkins J. M., Kligman D., James W. D. (2000). Neutrophilic dermatosis of the dorsal hands: Pustular vasculitis revisited. *Journal of the American Academy of Dermatology*.

[5] DiCaudo D. J., Connolly S. M. (2002). Neutrophilic dermatosis (pustular vasculitis) of the dorsal hands: A report of 7 cases and review of the literature. *Archives of Dermatology*.

[6] Larsen H., Danielsen A., Krustrup D., Weismann K. (2005). Neutrophil dermatosis of the dorsal hands. *Journal of the European Academy of Dermatology and Venereology*.

[7] Walling H. W., Snipes C. J., Gerami P., Piette W. W. (2006). The relationship between neutrophilic dermatosis of the dorsal hands and Sweet syndrome: Report of 9 cases and comparison to atypical pyoderma gangrenosum. *JAMA Dermatology*.

[8] Del Pozo J., Sacristán F., Martínez W., Paradela S., Fernández-Jorge B., Fonseca E. (2007). Neutrophilic dermatosis of the hands: Presentation of eight cases and review of the literature. *The Journal of Dermatology*.

[9] Paparone P. P., Paparone P. A., Senyatso R. Y. (2013). Neutrophilic dermatosis of the dorsal hand. *Wounds*.

[10] Cheng A. M., Cheng H. S., Smith B. J., Stewart D. A. (2018). Neutrophilic Dermatosis of the Hands: A Review of 17 Cases. *The Journal of Hand Surgery*.

[11] Wallach D. (2000). Neutrophilic dermatoses: An overview. *Clinics in Dermatology*.

[12] Bilu D., Kouba D. J., Mamelak A. J., Kazin R. A., Nousari C. H. (2004). Neutrophilic dermatosis of the dorsal hand. *The Journal of Dermatology*.

[13] Laguna C., Vilata J., Martín B. (2007). Neutrophilic Dermatosis of the Dorsal Hands. *Actas Dermo-Sifiliográficas*.

[14] Byun J. W., Hong W. K., Song H. J. (2010). A case of neutrophilic dermatosis of the dorsal hands with concomitant involvement of the lips. *Annals of Dermatology*.

[15] Malik M., Perkins W., Leach I. (2012). Anti-neutrophil cytoplasmic antibody-positive neutrophilic dermatosis of the dorsal hands. *Clinical and Experimental Dermatology*.

[16] Behrangi E., Rasi A., Attar B., Azizian Z. (2016). Neutrophilic dermatosis of dorsal hands and legs. *Archives of Iranian Medicine*.

[17] Weenig R. H., Bruce A. J., McEvoy M. T., Gibson L. E., Davis M. D. P. (2004). Neutrophilic dermatosis of the hands: Four new cases and review of the literature. *International Journal of Dermatology*.

[18] Hirai I., Sakiyama T., Konohana A., Takae Y., Matsuura S. (2015). A case of neutrophilic dermatosis of the dorsal hand in acute leukemia - a distributional variant of Sweet’s syndrome. *JDDG: Journal der Deutschen Dermatologischen Gesellschaft*.

[19] Fernández-Fernández F. J., Álvarez – Fernández J. C., Romero – Picos E., Garrido J. A., Sesma P. (2010). Neutrophilic Dermatosis of the Dorsal Hands Associated with a “Myeloproliferative Neoplasm, Unclassifiable“ and a Simultaneous Cancer of Colon. *Acta Medica (Hradec Kralove)*.

[20] Gonzalez A., Vaziri S., Brandt J. C., Steffes W., Perbtani Y. (2016). Neutrophilic dermatosis of the dorsal hands in an elderly man. *Dermatology Online Journal*.

[21] Cravo M., Cardoso J. C., Tellechea O., Cordeiro M. R., Reis J. P., Figueiredo A. (2008). Neutrophilic dermatosis of the dorsal hands associated with hypopharyngeal carcinoma. *Dermatology Online Journal*.

[22] Leecy T., Anderson A., Von Nida J., Harvey N., Wood B. (2013). Neutrophilic dermatosis of the dorsal hands: An often under recognised and mistreated entity. *Pathology*.

[23] Brajon D., Cuny J.-F., Barbaud A., Schmutz J.-L. (2011). Neutrophilic dermatosis of the hands. *Annales de Dermatologie et de Venereologie*.

[24] Benzimra J., Low-Beer J., Twomey J. (2011). A case of peripheral ulcerative keratitis associated with neutrophilic dermatosis of the dorsal hand. *International Ophthalmology*.

[25] Kaur S., Gupta D., Garg B., Sood N. (2015). Neutrophilic dermatosis of dorsal hands. *Indian Dermatology Online Journal (IDOJ)*.

[26] Baz K., Yazici A. C., Kaya T. I. (2003). Neutrophilic dermatosis of the hands (localized Sweet's syndrome) in association with chronic hepatitis C and sarcoidosis. *Clinical and Experimental Dermatology*.

[27] Aydin F., Senturk N., Yildiz L., Canturk M. T., Turanli A. Y. (2004). Neutrophilic dermatosis of the dorsal hands in a farmer. *Journal of the European Academy of Dermatology and Venereology*.

[28] Thatte S., Aggarwal A. (2015). Neutrophilic dermatosis of the dorsal hands: A rare unilateral presentation. *Indian Dermatology Online Journal (IDOJ)*.

[29] Mathieu S., Soubrier M., Tournadre A., Dubost J.-J. (2014). Neutrophilic dermatosis of the dorsal hands during thalidomide treatment. *International Journal of Dermatology*.

[30] Hoverson A. R., Davis M. D. P., Weenig R. H., Wolanskyj A. P. (2006). Neutrophilic dermatosis (sweet syndrome) of the hands associated with lenalidomide. *Archives of Dermatology*.

[31] Rockers K. M., Fielder L. M. (2009). Neutrophilic dermatosis of the dorsal hands in a patient treated with chemotherapy for acute myelogenous leukemia. *Cutis*.

[32] Cook E., Epstein R., Miller R. (2011). A rare case of idiopathic neutrophilic dermatosis of the hands. *Dermatology Online Journal*.

